# Efficacy and safety of percutaneous transcatheter aortic valvuloplasty prior to non-cardiac surgery in Japanese patients with severe aortic stenosis

**DOI:** 10.1007/s12928-019-00581-1

**Published:** 2019-03-07

**Authors:** Hiroya Takafuji, Shinobu Hosokawa, Riyo Ogura, Yoshikazu Hiasa

**Affiliations:** Department of Cardiology, Tokushima Red Cross Hospital, 103 Irinokuchi, Komatsushima-cho, Komatsushima, Tokushima 773-8502 Japan

**Keywords:** Percutaneous transcatheter aortic valvuloplasty, Severe aortic stenosis, Non-cardiac surgery

## Abstract

This study aimed to investigate the efficacy of percutaneous transcatheter aortic valvuloplasty (PTAV) performed prior to non-cardiac surgery and the safety of non-cardiac surgery after PTAV in elderly Japanese patients. Between March 2012 and August 2018, 14 patients who underwent PTAVs prior to non-cardiac surgery were enrolled. The mean age was 82.2 ± 7.0 years. A total of 9 patients (64.3%) were women. A retrograde approach was selected for 57.1% of the patients. More than 75% of the procedures were performed using echocardiographic imaging. Echocardiographic data including the aortic valve area (AVA), peak aortic valve blood velocity flow (AVF), peak aortic valve pressure gradient (AVPG), and mean AVPG significantly improved after PTAV (AVA; from 0.54 ± 0.11 to 0.80 ± 0.13 cm^2^, peak AVF; from 4.6 ± 0.8 to 3.8 ± 0.7 m/s, peak AVG; from 87.9 ± 28.0 to 62.2 ± 19.9 mmHg, mean AVG; from 49.8 ± 18.9 to 35.7 ± 11.6 mmHg; *p* < 0.001, *p* < 0.001, *p* < 0.001, *p* = 0.0012, respectively). Neither complications related to the PTAV procedure nor procedural mortality were noted. Non-cardiac surgery after PTAV was safely performed; there were no significant adverse events during non-cardiac surgery and no in-hospital mortality occurred after non-cardiac surgery. PTAV prior to non-cardiac surgery in elderly Japanese patients with severe aortic stenosis is safe and effective. In addition, non-cardiac surgery after PTAV can be safety performed without adverse events.

## Introduction

Elderly patients undergoing non-cardiac surgery incidentally have severe aortic stenosis (AS). The current Japanese Circulation Society Guidelines indicate that patients undergoing non-cardiac surgery with symptomatic severe AS should be treated prior to non-cardiac surgery [1]. In the guidelines, echocardiography is recommended for patients with asymptomatic severe AS before non-cardiac surgery to determine cardiac function and the degree of invasion of the non-cardiac surgery. Similarly, the current European Society of Cardiology (ESC)/the European Society of Anaesthesiology (ESA) guidelines recommend that aortic valve replacement (AVR) should be considered in asymptomatic patients with severe AS, or with Class IIa, who are scheduled for elective high-risk non-cardiac surgery [2]. However, it is difficult to determine whether patients are asymptomatic or not based on the values of the elderly population in Japan, as they are more likely to hide their symptoms. In addition, it is difficult to wait for non-cardiac surgery until AVR or transcatheter aortic valve implantation (TAVI), as the waiting period for these treatments differs among institutions. Moreover, it is commonly known that anesthesia for patients with severe AS induces several perioperative risks, such as hypotension and reduced coronary perfusion, leading to myocardial ischemia and a downward spiral of reduced contractility [3]. Especially in elderly patients with severe AS, the number and severity of comorbidities are generally higher than in patients without severe AS. On the other hand, percutaneous transcatheter aortic valvuloplasty (PTAV) could temporarily improve the severity of AS by performing minimally invasive treatment for elderly patients before non-cardiac surgery. The aim of the present study was to investigate the efficacy of PTAV prior to non-cardiac surgery and the safety of non-cardiac surgery after PTAV in Japanese patients with severe AS.

## Methods

### Study population

**Fig. 1:**
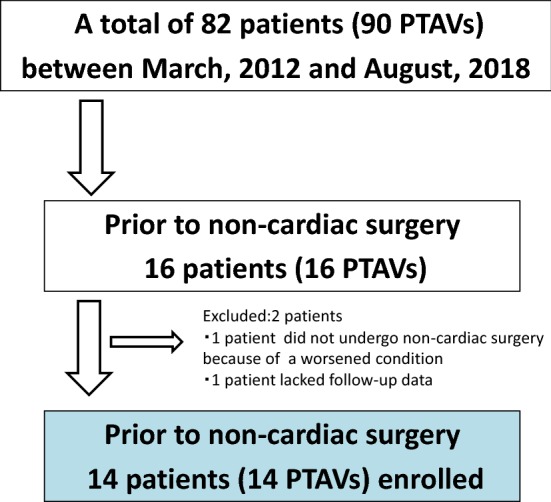
Flowchart of the present study. *PTAV* percutaneous transcatheter aortic valvuloplasty

The present study was a single-center, retrospective study. Between March 2012 and August 2018, a total of 90 PTAV procedures in 82 patients with severe AS were performed at Tokushima Red Cross Hospital in Japan. Of these 82 patients, 16 patients (19.5%) underwent PTAV prior to non-cardiac surgery. Indication of PTAV prior to non-cardiac surgery was determined at the heart team conference. This conference included the anesthetist, the surgeon, and the cardiovascular interventionist. Decisions were made taking into account operative risk, comorbidities, frailty, and the urgency of non-cardiac surgery. A flowchart of this study is shown in Fig. 1. Two patients were excluded: one patient did not undergo non-cardiac surgery because of a worsened condition, and the other patient lacked follow-up data. Finally, 14 patients were enrolled. We evaluated the patients’ characteristics, procedural characteristics, change of echocardiographic parameters, and outcomes.

### Definitions

Severe AS was defined as an aortic valve area (AVA) < 1 cm^2^ and/or mean aortic valve pressure gradient (AVPG) ≥ 40 mmHg and/or peak aortic valve blood velocity flow (AVF) ≥ 4 m/s according to transthoracic echocardiography (TTE). Symptoms of severe AS were defined as chest pain, syncope, dyspnea, and a history of heart failure.

### Percutaneous transcatheter aortic valvuloplasty (PTAV)

PTAV was performed in a hybrid operation room or catheterization room. The PTAV procedure was performed under the conventional technique. The decision of approach (antegrade or retrograde) and anesthesia (general or local) during PTAV was independently determined by operators. The antegrade approach was performed by using the INOUE balloon catheter (Toray Industries, Inc., Tokyo, Japan). The retrograde approach was performed by using the Maxi-LD balloon catheter (CardinalHealth Inc., Dublin, OH, USA), TYSHAK balloon catheter (NuMED Inc., Hopkinton, NY, USA), ZMED balloon catheter (NuMED Inc., Hopkinton, NY, USA), or TMP BAV balloon catheter (Tokai Medical Products, Aichi, Japan). The balloon size was determined according to the annular diameter of the patients, using computed tomography (CT) or TTE. CT and TTE can be used in conjunction with or be replaced by perioperative transesophageal echocardiography as well as perioperative intracardiac echocardiography (ICE). The balloon was dilated step by step upsizing, increasing the size to the largest annular diameter measured without causing worsened aortic regurgitation (AR). The desired end point of the procedure was the achievement of a mean AVPG < 40 mmHg or a calculated AVA over 1 cm^2^.

### Statistical analysis

Statistical analysis was performed using JMP version 8 (SAS Institute Inc, Cary, NC, USA). Continuous variables are expressed as mean ± standard deviation. Categorical variables were compared between the groups using the Chi squared test or Fisher’s exact test. Values of *p* < 0.05 were considered statistically significant.

## Results

Fourteen patients who underwent PTAV prior to non-cardiac surgery between March 2012 and August 2018 were analyzed. Patients’ data were retrospectively collected by electronic medical records.

### Distribution of non-cardiac surgery

**Fig. 2:**
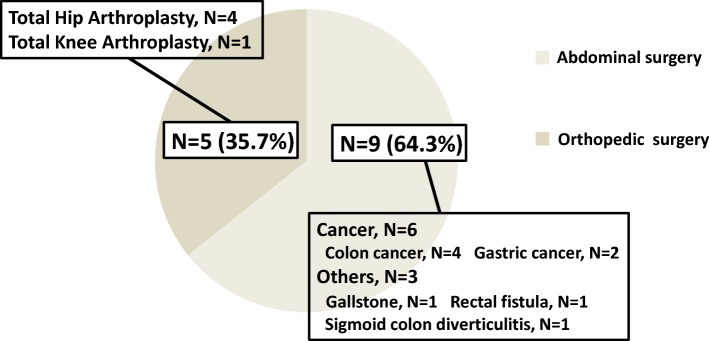
Distribution of non-cardiac surgery

The distribution of non-cardiac surgery is shown in Fig. 2. Nine of the 14 patients (64.3%) underwent abdominal surgery. Five of the 14 patients (35.7%) underwent orthopedic surgery. Of those who underwent abdominal surgery, 6 of 9 cases involved cancer (4 patients had colon cancer and 2 patients had gastric cancer). All orthopedic surgeries involved open reduction of fractures.

### Patients’ characteristics

Patients’ characteristics are listed in Table 1. The mean age was 82.2 ± 7.0 years. A total of 9 patients (64.3%) were women. The prevalence of hypertension was high (71.4%). More than half of the patients (57.1%) had chronic kidney disease. Mean brain natriuretic peptide (BNP) was 336 ± 532 pg/ml and mean systolic pulmonary artery pressure (PAP) by TTE was 33.8 ± 9.6 mmHg. According to the New York Heart Association (NYHA) functional class, a total of 3 (21.8%) had a class III rating and no patients were class IV. A history of heart failure was found in 28% of the patients. No symptoms of AS were found in 9 patients (64.3%). The mortality risk, according to the Society of Thoracic Surgeons (STS) score, was 4.195 ± 1.879%.

**Table 1:** Baseline patients’ characteristics

Characteristics	Overall (*n* = 14)
Age (year)	82.2 ± 7.0
Female gender (%)	9 (64.3)
BMI (kg/m^2^)	22.2 ± 2.9
Smoking (%)	5 (35.7)
Hypertension (%)	10 (71.4)
Diabetes mellitus (%)	5 (35.7)
Dyslipidemia (%)	5 (35.7)
Chronic kidney disease (%)	8 (57.1)
Hemodialysis (%)	0 (0)
Previous MI (%)	1 (7.1)
Previous PCI (%)	2 (14.3)
Previous CABG (%)	0 (0)
Old cerebral infarction (%)	3 (21.4)
BNP (pg/ml)	336 ± 532
Systolic PAP (mmHg)	33.8 ± 9.6
NYHA functional class	
III (%)	3 (21.4)
IV (%)	0 (0)
Previous heart failure (%)	4 (28.6)
Syncope (%)	1 (7.1)
Angina (%)	1 (7.1)
Asymptomatic (%)	9 (64.3)
STS score	4.195 ± 1.879

Values represent the mean ± SD or *n* (%)

*BMI* body mass index, *MI* myocardial infarction, *PCI* percutaneous coronary intervention, *BNP* brain natriuretic peptide, *PAP* pulmonary artery pressure, *NYHA* New York Heart Association, *STS* the Society of Thoracic Surgeons

### Procedural characteristics of PTAV

The procedural characteristics are detailed in Table 2. Procedural time of PTAV was 83.2 ± 22.1 min. The retrograde approach was selected in 57.1% and the mean inflation balloon size used was 20.5 ± 2.8 mm (15–25 mm). Eleven (78.6%) of the total procedures were performed using an echocardiographic imaging guide, such as TEE and ICE. More than half of the procedures (64.3%) were performed under general anesthesia.

**Table 2:** Procedural characteristics

Characteristics	Overall (*n* = 14)
Procedural time (min)	83.2 ± 22.1
Approach	
Antegrade approach (%)	6 (42.9)
Retrograde approach (%)	8 (57.1)
Maximum balloon size (mm)	20.5 ± 2.8
Range (mm)	15–25
Number of balloon inflations	4.4 ± 3.4
Imaging guide	
TEE or/and ICE guide (%)	11 (78.6)
Fluoroscopic guide (%)	3 (21.4)
Anesthesia	
General anesthesia (%)	9 (64.3)
Local anesthesia (%)	5 (35.7)

Values represent the mean ± SD or *n* (%)

*TEE* transesophageal echocardiography, *ICE* intracardiac echocardiography

### Catheter-derived hemodynamic measurements and echocardiographic data before and after PTAV

Catheter-derived hemodynamic measurements and echocardiographic data before and after PTAV are shown in Table 3. The AVA according to the Gorlin equation and mean AVPG with simultaneous catheter measurement were significantly improved (from 0.56 ± 0.15 to 0.83 ± 0.12 cm^2^, from 56.9 ± 20.7 to 34.3 ± 13.6 mmHg, *p* < 0.001, *p* < 0.001, respectively). TTE was performed at baseline (mean 5.7 ± 5.4 days before PTAV) and immediately following the procedure (mean 1.2 ± 0.6 days after PTAV). The AVA, peak AVF, peak AVPG, and mean AVPG significantly improved (from 0.54 ± 0.11 to 0.80 ± 0.13 cm^2^, from 4.6 ± 0.8 to 3.8 ± 0.7 m/s, from 87.9 ± 28.0 to 62.2 ± 19.9 mmHg, from 49.8 ± 18.9 to 35.7 ± 11.6 mmHg, *p* < 0.001, *p* < 0.001, *p* < 0.001, *p* = 0.0012, respectively). No statistically significant differences in left ventricular ejection fraction (LVEF), left ventricular diameter at end diastole (LVDd), left ventricular diameter at end systole (LVDs), and severity of aortic regurgitation (AR) were noted. Outcomes are shown in Table 4.

**Table 3:** Catheter and echocardiographic data before and after percutaneous transcatheter aortic valvuloplasty

Variable	Before PTAV (*n* = 14)	After PTAV (*n* = 14)	*p* value
Catheter data			
AVA (cm^2^)	0.56 ± 0.15	0.83 ± 0.21	*p* < 0.001
Mean AVPG (mmHg)	56.9 ± 20.7	34.3 ± 13.6	*p* < 0.001
Echocardiographic data			
AVA (cm^2^)	0.54 ± 0.11	0.80 ± 0.13	*p* < 0.001
Peak AVF (m/s)	4.6 ± 0.8	3.8 ± 0.7	*p* < 0.001
Peak AVPG (mmHg)	87.9 ± 28.0	62.2 ± 19.9	*p* < 0.001
Mean AVPG (mmHg)	49.8 ± 18.9	35.7 ± 11.6	0.0012
LVEF (%)	63.6 ± 12.4	66.7 ± 10.9	0.16
LVDd (mm)	44.6 ± 8.4	45.3 ± 8.5	0.56
LVDs (mm)	29.4 ± 8.9	28.7 ± 8.2	0.48
AR ≧ moderate (%)	1 (7.1)	1 (7.1)	1.00

Values represent the mean ± SD or *n* (%)

*PTAV* percutaneous transcatheter aortic valvuloplasty, *AVA* aortic valve area, *AVF* aortic valve blood velocity flow, *AVPG* aortic valve pressure gradient, *LVEF* left ventricular ejection fraction, *LVDd* left ventricular diameter at end diastole, *LVDs* left ventricular diameter at end systole, *AR* aortic regurgitation

**Table 4:** Outcomes of percutaneous transcatheter aortic valvuloplasty and non-cardiac surgery

Variable	Overall (*n* = 14)
Procedural complication (%)	0 (0)
Annulus rupture (%)	0 (0)
Cardiac tamponade (%)	0 (0)
Major bleeding needing transfusion (%)	0 (0)
Arrhythmia (%)	0 (0)
Procedural mortality (%)	0 (0)
Heart failure after PTAV until discharge (%)	0 (0)
Days between PTAV and non-cardiac surgery (days)	8.4 ± 3.9
Procedure of non-cardiac surgery	
Open surgery	1 (7.1)
Laparoscopy surgery	8 (57.1)
Orthopedic surgery	5 (35.7)
Anesthesia during non-cardiac surgery	
General anesthesia (%)	13 (92.9)
Inhalation anesthesia (%)	10 (71.4)
TIVA (%)	2 (14.3)
Inhalation anesthesia and TIVA (%)	1 (7.1)
Local anesthesia (%)	1 (7.1)
Spinal anesthesia (%)	1 (7.1)
Procedural time of non-cardiac surgery (min)	108.3 ± 65.8
Anesthesia time of non-cardiac surgery (min)	153.6 ± 69.5
Significant adverse event during non-cardiac surgery (%)	0 (0)
In-hospital mortality after non-cardiac surgery (%)	0 (0)

Values represent the mean ± SD or *n* (%)

*PTAV* percutaneous transcatheter aortic valvuloplasty, *TIVA* total intravenous anesthesia, *AVR* aortic valve replacement, *TAVI* trans catheter aortic valve implantation

### Outcomes

There were no complications related to the procedure, such as annulus rupture, cardiac tamponade, major bleeding, and arrhythmia. All cases achieved procedural success and no procedural mortality occurred. Non-cardiac surgery was performed 8.4 ± 3.9 days after PTAV. Almost all abdominal surgeries were performed laparoscopically and all orthopedic surgeries were performed for open reduction of fractures. A total of 13 (92.9%) non-cardiac surgeries were performed under general anesthesia, especially using inhalation anesthesia. There were no significant adverse events during non-cardiac surgery and no in-hospital mortalities after non-cardiac surgery.

## Discussion

This study shows that PTAV before non-cardiac surgery for elderly Japanese patients with severe AS is safe and effective, and that non-cardiac surgery after PTAV can be safely performed without adverse events.

AS is the most common valvular disease among the elderly population. A previous study has reported that the prevalence of AS and severity of AS in the elderly (age ≥ 75 years) was 12.5% and 3.4%, respectively [4]. In addition, 5–7% of the elderly population over 80 years old and 10–15% over 90 years old have severe AS [5, 6]. The survival duration of patients with severe AS without intervention is 1.8 years [7]. Accordingly, the current guidelines recommend surgical AVR or TAVI for symptomatic severe AS without severe comorbidities [8, 9]. On the other hand, the symptoms of AS are not clearly determined among elderly Japanese patients because most patients easily tolerate the symptoms. In this study, 64.9% of patients showed no symptoms of severe AS. Moreover, it is well known that management of patients with severe AS during non-cardiac surgery is difficult. Excessive dehydration is related to cardiovascular collapse; in addition, volume overload causes heart failure. Therefore, anesthesiologists generally hope that the severity of AS improves before non-cardiac surgery, if possible.

PTAV was first reported by Alain Cribier in 1986 as a less invasive treatment for severe AS in elderly and/or high-risk patients [10, 11]. It is now a global procedure because it was an option for treatment of patients with severe AS. However, the efficacy of PTAV is limited and intermittent. One previous report shows a decrease in AVA at 6 months and 12 months (both − 0.19 cm^2^, *p* < 0.05) and an increase in pAVG at 6 months (+ 19.9 mmHg, *p* < 0.05) and 12 months (+ 23.8 mmHg, *p* < 0.05) as compared to values after PTAV [12]. A few other reports showed that hemodynamic conditions, such as cardiac output, immediately improve after PTAV [13, 14]. Furthermore, in the TAVI era, an indication for treatment of PTAV is mainly palliative care or temporary improvement of AS severity. Safe anesthesia is required to undergo non-cardiac surgery. Although the severity of AS improves temporarily after PTAV, it has been proved to be effective for non-cardiac surgery. TAVI and surgical AVR are well-known options to improve cardiac symptoms prior to non-cardiac surgery. However, it is recommended to prescribe antiplatelet or anticoagulation therapy following these procedures. In general, both surgeons and anesthetists are reluctant to use antiplatelet and anticoagulation therapy due to the increased risk of hemorrhage. PTAV prior to non-cardiac surgery is beneficial because it can be performed without these medications. Furthermore, it is difficult for patients to wait for non-cardiac surgery until TAVI or AVR has been performed. TAVI or AVR requires anatomical abnormalities to be ruled out, using modalities such as CT, magnetic resonance imaging (MRI), and coronary angiography. PTAV can be performed without confirming anatomical information. In addition, elderly patients are generally frail. More than half the patients in this study (64.3%) did not receive TAVI or AVR following non-cardiac surgery due to a combination of frailty and multiple comorbidities. In this study, we considered whether there is any difference in thoroughness in performing PTAV before non-cardiac surgery, as compared to performing PTAV as a palliative procedure. In other words, modest hemodynamic and anatomical improvements over PTAV are regarded as acceptable end point in cases of prior non-cardiac surgery preparation PTAV. PTAV prior to non-cardiac surgery is preparation for the subsequent procedure; the main problem that these patients have is the one which requires treatment by non-cardiac surgery. Therefore, we did not aggressively pursue improvements in AVA or mean AVPG based on the decision made by our heart team. It is more important for non-cardiac patients that PTAV is performed safely, without complications such as acute AR or annular rupture. As a result, the severity of AS in patients in the present study was improved from a severe grade to a moderate grade. Although this was not a complete treatment of severe AS, patients treated by pre-non-cardiac surgery PTAV safely underwent non-cardiac surgery without any peri-surgical cardiac events.

This study has several limitations. First, this analysis was retrospective and non-randomized in design; therefore, there was no comparison between non-cardiac surgery with PTAV and without PTAV. Second, this study was conducted at a single center, and the patient sample size was small. Third, our study population of patients who underwent non-cardiac surgery was limited only to abdominal and orthopedic surgeries. Furthermore, almost all patients had asymptomatic severe AS. However, in the clinical setting, elderly Japanese patients sometimes were not clear regarding how noticeable their AS symptoms were. Although further investigation will be needed to validate our findings, we suggest that PTAV prior to non-cardiac surgery is safe and effective in elderly Japanese patients with severe AS.

## Conclusion

PTAV prior to non-cardiac surgery in elderly Japanese patients with severe AS is safe and effective. In the TAVI era, PTAV for severe AS should be considered as an option to improve the patient’s hemodynamic status before non-cardiac surgery.
